# Editorial: Cell-to-cell communications in tissue homeostasis and repair

**DOI:** 10.3389/fcell.2023.1138621

**Published:** 2023-01-13

**Authors:** Osamu Shimmi, Esko Kankuri

**Affiliations:** ^1^ Institute of Biotechnology, University of Helsinki, Helsinki, Finland; ^2^ Institute of Molecular and Cell Biology, University of Tartu, Tartu, Estonia; ^3^ Department of Pharmacology, Faculty of Medicine, University of Helsinki, Helsinki, Finland

**Keywords:** endometrium, wound healing, regeneration, psoriasis, dentinogenesis, epithelial morphogenesis

Increased understanding of the intricate networks and signaling that drive organ and tissue development ultimately translate into therapeutic benefit and successes such as the ability to induce adult somatic cell pluripotency and controlling the rampant proliferation of cancer cells by sophisticated biological drugs. Moreover, rationally engineered therapeutics can help us drive functional tissue regeneration, modify or reverse the courses of disease processes and gain more targeted control over dysregulated immune reactivity.

With this Research Topic, our aim was to attract colleagues to share their work on the various aspects of cell signaling and to share exciting information on its contribution to the physiological, pathological, and therapeutic mechanisms. We are grateful for all the contributing authors for sharing their work and results to add to our understanding of the vital process of cell-cell communication. The insights provided in the five papers of this Research Topic provide important information ranging from tissue development and regulation of cell proliferation to identification of endogenous pathways and targets for regenerative tissue therapies as well as for promoting wound healing and controlling chronic immunological activation.

In their report, Huang et al. investigated the roles of Scribble (Scrib), a key apicobasal cell polarity determinant, in the control of epithelial homeostasis and growth. The authors first demonstrate the interaction of Scrib with α-Catenin (α-Cat), one of the core components of adherens junctions, in the regulation of YAP, an effector of the Hippo signaling pathway, in human epithelial cells. They then utilize the *Drosophila* wing imaginal disc to show an inverse correlation between *scrib* expression and Yki activity, an ortholog of YAP in flies, as well as wing size control by quantity of Scrib. Finally, the authors show that the elimination of *scrib* mutant cells in the context of cell competition can be rescued by the expression of a fusion protein of Scrib-LRR and α-Cat. These facts, together with the authors’ recent findings in flies (Gui et al., 2021), reveal that the association of Scrib with α-Cat plays a conserved role in epithelial homeostasis and growth.

The study by Fang et al. provides a rich compendium recourse of gene expression profiles of cell-cell interactome among endometrial cells. This paper describes a single cell transcriptomic study of proliferative phase endometrium biopsies from three healthy women. The authors obtained patterns of cell-cell communication by focusing on families of signal transduction pathways. The CellChat software, a versatile and easy-to-use toolkit for inferring, analyzing, and visualizing cell-cell communication from any given scRNA-seq data (Jin et al., 2021), was used for their communication network analysis. Cell interactions are part of the core biological function of endometrial tissues (Hernandez and Iruela-Arispe 2020), and the authors’ report demonstrates a successful utilization of the CellChat communication pattern analysis to uncover coordinated responses among different endometrial cell types.

The study by Duncan et al. used matrix-metalloproteinase-13 knockout mice (*Mmp13−/−*) to analyze MMP13-dependent phenotypic changes in the dentin-pulp complex, mineralization-associated marker expression and associated mechanistic interactions. The authors first show that MMP13 is highly expressed in the dental tissue during development. *Mmp13* deletion affects dental pulp cell proliferation during tooth development and the expression of ameloblastic, odontoblastic, and mineralization-associated gene and protein markers. The paper also reveals mechanistic interactions of class IIa HDACs and Wnt signaling for the physiological function of MMP13 in developing teeth. The results indicate that MMP13 plays an important role in multiple functions critical to the regulation of tooth development, odontogenic differentiation, and dentin-pulp reparative mechanisms.

Two papers of the Research Topic addressed disease processes of the skin. Reciprocal signaling and crosstalk between the dermal and epidermal compartments of the skin is essential for physiological maintenance of homeostasis as well as activation of wound healing pathways upon injury. In their paper, Klaas et al. provide insights to the selectivity of stimuli arising from the dermal matricellular compartment after skin injury and during chronic inflammation. The authors focused on thrombospondin-4 (THBS4), a matricellular glycoprotein, which binds to the extracellular matrix (ECM) and signals through a number of cellular receptors (Murphy-Ullrich and Iozzo 2012). THBS4 signaling shows a high level of dynamic variability through interactions with growth factors, such a transforming growth factor-beta, ECM ligands (such as collagens I–III and V), heparan sulphates and cell surface integrins (Stenina-Adognravi and Plow 2019). THBS4 is highly expressed during embryonal development, and while its expression is low in adult tissues after injury it is expressed for formation of a transitional matrix important in ECM remodeling (Stenina-Adognravi and Plow 2019). In samples from burn wound patients, the expression of THBS4 is increased after wounding and during the early stages of wound healing. The authors also demonstrate that THBS4 can exert differential cell-type-dependent responses promoting the proliferation of human keratinocytes and the migration of fibroblasts, and that topical supplementation of recombinant THBS4 acts therapeutically to promote experimental wound healing *in vivo*. Moreover, the paper shows that THBS4 expression is increased in psoriasis, characterized with chronic inflammation and keratinocyte hyperproliferation. Interestingly, in psoriatic lesions THBS4 expression was localized to the upper papillary dermis and basal epidermis—suggesting that the hyperproliferative keratinocyte response of psoriasis may also involve dysregulated THBS4 signaling. Recently, the authors have expanded their findings and have identified pathways activated by THBS4 in keratinocytes to converge with those involved in the pathogenesis of atopic dermatitis (Mäemets-Allas et al., 2022).

Zang et al. provide an alternate view into psoriasis therapeutics by employing small extracellular vesicles (sEVs) from mesenchymal stem cells (MSCs). The authors first demonstrate that sEVs from human umbilical-cord-derived MSCs stimulated by IFN-gamma (IFNgamma-sEVs), inhibit T cell proliferation and differentiation *in vitro* and *in vivo*. They then loaded these IFNgamma-sEVs with oligonucleotides against miR-210 (ASO-210), which has been identified as an inflammation-driving factor in psoriasis (Wu et al., 2018). Treatment with ASO-210-loaded IFNgamma-sEVs exerted a stronger protective effect against psoriatic inflammation than a similar dose of ASO-210 and IFNgamma-sEVs administered as a mixture. The results reveal the therapeutic potential of MSC-sEVs as active immunosuppressant delivery vehicles of oligonucleotide therapeutics for psoriasis treatment.

We are grateful to all contributors for selecting this forum for these excellent scientific works and hope that this Research Topic, for its part, thrusts the field forward to open new insights in cell and developmental biology.
